# Meet Me in the Middle: Median Temperatures Impact Cyanobacteria and Photoautotrophy in Eruptive Yellowstone Hot Springs

**DOI:** 10.1128/msystems.01450-21

**Published:** 2022-01-04

**Authors:** Trinity L. Hamilton, Jeff Havig

**Affiliations:** a Department of Plant and Microbial Biology, University of Minnesota, St. Paul, Minnesota, USA; b The Biotechnology Institute, University of Minnesota, St. Paul, Minnesota, USA; c Department of Earth and Environmental Sciences, University of Minnesota, Minneapolis, Minnesota, USA; University of California San Diego

**Keywords:** hot springs, photoassimilation, phototroph, oxygenic photosynthesis, *Chloroflexi*, aerobic anoxygenic phototroph, pH, temperature, *Cyanobacteria*, *Chlorobi*, *Synechococcus*, geyser

## Abstract

Geographic isolation can be a main driver of microbial evolution in hot springs while temperature plays a role on local scales. For example, cyanobacteria, particularly high-temperature *Synechococcus* spp., have undergone ecological diversification along temperature gradients in hot spring outflow channels. While water flow, and thus temperature, is largely stable in many hot springs, flow can vary in geysing/eruptive hot springs, resulting in large temperature fluctuations (sometimes more than 40°C). However, the role of large temperature fluctuations in driving diversification of cyanobacteria in eruptive hot springs has not been explored. Here, we examined phototroph community composition and potential photoautotrophic activity in two alkaline eruptive hot springs with similar geochemistry in the Lower Geyser Basin in Yellowstone National Park, WY. We observed distinct cyanobacterial amplicon sequencing variants (ASVs) consistent with allopatry and levels of light-dependent inorganic carbon uptake rates similar to other hot springs, despite large temperature fluctuations. Our data suggest median temperatures may drive phototroph fitness in eruptive hot springs while future studies are necessary to determine the evolutionary consequences of thriving under continuously fluctuating temperatures. We propose that large temperature swings in eruptive hot springs offer unique environments to examine the role of allopatry versus physical and chemical characteristics of ecosystems in driving cyanobacterium evolution and add to the debate regarding the ecology of thermal adaptation and the potential for narrowing niche breadth with increasing temperature.

**IMPORTANCE** Hot spring cyanobacteria have long been model systems for examining ecological diversification as well as characterizing microbial adaptation and evolution to extreme environments. These studies have reported cyanobacterial diversification in hot spring outflow channels that can be defined by distinct temperature ranges. Our study builds on these previous studies by examining cyanobacteria in geysing hot springs. Geysing hot springs result in outflow channels that experience regular and large temperature fluctuations. While community compositions are similar between geysing and nongeysing hot spring outflow channels, our data suggest median, rather than high, temperature drives the fitness of cyanobacteria in geysing hot springs. We propose that large temperature swings may result in patterns of ecological diversification that are distinct from more stable outflows.

## OBSERVATION

**FIG 1:**
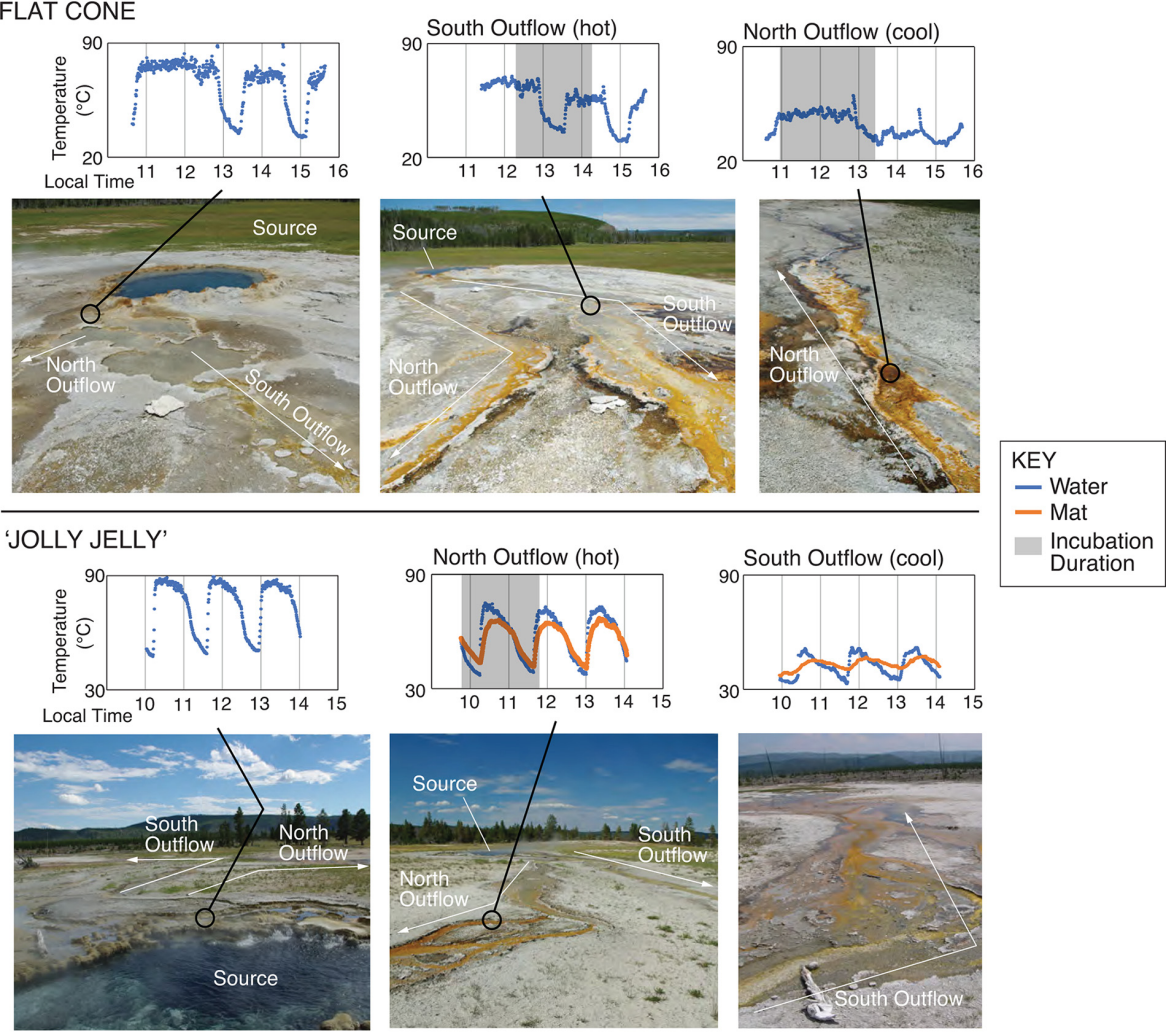
Site photos and temperature variation. (Top) Temperature measured over a 4-h window near the FC source and two outflow locations: FC hot (where phototrophs were first visible in the center of the outflow channel) and FC cool. (Bottom) Temperature measured over a 4-h window near the JJ source and two outflow locations: JJ hot (where phototrophs were first visible in the center of the outflow channel) and JJ cool. Both sites are located in the Lower Geyser Basin in YNP (Fig. S1). Site information (location and select physical and geochemical measurements) is provided in Table S1.

Cyanobacteria in hot springs tend to form geographically isolated populations (1, 2) while outflow channel temperature gradients can select for highly adapted, ecologically distinct populations (ecotypes) (1, 2). For example, *Synechococcus* ecotypes are structured by temperature along the stable flow outflow channels of Mushroom and Octopus Springs in the Lower Geyser Basin (LGB) of Yellowstone National Park (YNP), WY, USA (3–8). In contrast, geysing hot spring outflow channels undergo large temperature fluctuations due to eruptive cycles: continuous flow, a temperature spike from an acute eruption, and a no-flow period during source recharge. For the ∼500 geysing hot springs in YNP (9, 10), eruption periodicities range from regular (e.g., Old Faithful is ∼91 to 93 min) to chaotic (e.g., Steamboat Geyser can vary from 3 days to 50 years [11]). Here, we examined phototrophic community composition coupled to rates of light-dependent C assimilation (via ^13^C-labeled bicarbonate microcosms) in the outflow channels of two eruptive hot springs with similar geochemical profiles (12) (see Table S1 in the supplemental material): Flat Cone (FC) and an unnamed feature we colloquially named “The Jolly Jelly” (JJ; YNP Thermal Feature Inventory ID LFMNN010) in LGB, YNP (Fig. 1; Fig. S1).

10.1128/msystems.01450-21.2 FIG S1 Sampling locations. (Upper left) Region of the United States that includes the Yellowstone National Park area. Inset box “A” indicates the region sampled within the Lower Geyser Basin, Yellowstone National Park, USA. (Upper right) (A) Western portion of the Lower Geyser Basin including Sentinel Meadows (upper left portion of image) and the Imperial Geyser Basin (bottom center of image). Inset box “B” indicates area of “The Jolly Jelly,” and inset box “C” indicates area of Flat Cone. (Bottom left) (B) “The Jolly Jelly.” (Bottom right) (C) Flat Cone. Images provided courtesy of Google Earth. Download FIG S1, PDF file, 0.3 MB. Copyright © 2022 Hamilton and Havig. 2022 Hamilton and Havig. https://creativecommons.org/licenses/by/4.0/ This content is distributed under the terms of the Creative Commons Attribution 4.0 International license.

10.1128/msystems.01450-21.5 TABLE S1 GPS coordinates, pH, conductivity, temperature, and aqueous geochemistry of spring water at the site of sample collection and microcosm incubations. Dissolved inorganic carbon (DIC), DIC δ^13^C values, dissolved organic carbon (DOC), and DOC δ^13^C values are also provided. pH and temperature were recorded at the time of sample collection. Sulf, sulfide; bdl, below detection limit. Detection limits: nitrate, 0.01 mg/L NO_3_^−^; Fe^2+^, 20 μg/L. Download Table S1, PDF file, 0.09 MB. Copyright © 2022 Hamilton and Havig. 2022 Hamilton and Havig. https://creativecommons.org/licenses/by/4.0/ This content is distributed under the terms of the Creative Commons Attribution 4.0 International license.

FC exhibits a more chaotic eruption periodicity—106 min on average, ranging from 25 min to >12 h—but maintains a steady temperature/outflow rate ∼68% of the time (Fig. 1; Fig. S2). JJ exhibits a more regular eruption periodicity—88 min on average, ranging from 76 to 103 min (13), with a continuous but fluctuating discharge ∼54% of the time (Fig. 1; Fig. S3). At FC, phototrophs were first visible in the center of the south outflow channel ∼8 m from the source (here designated “FC hot”). Temperatures at FC hot varied by 40.5°C during a 4-h observation period: median of 56.0°C, with maximum of 70.0°C and minimum of 29.5°C (Fig. 1). Downstream from the photosynthetic fringe (∼14 m from the source, here designated “FC cool”), water reached a median of 40.0°C over a 4-h period (maximum = 60.0°C, minimum = 29.0°C). At JJ, phototrophs were first visible in the center of the north outflow channel ∼24 m from the source (here designated “JJ hot”). At JJ hot, temperatures varied by 38.0°C during a 4-h observation period: median of 61.5°C, with maximum of 75.0°C and minimum of 37.0°C (Fig. 1). Further downstream (∼60 m from the source, here designated “JJ cool”), the median was 42.5°C (maximum of 52.0°C, minimum of 33.0°C). Temperatures deeper in the phototrophic mats were muted compared to that of the water at the mat-water interface (Fig. 1): at a depth of ∼1 cm in the JJ hot mats, the median was 58.5°C, with maximum of 67.5°C and minimum of 40.5°C.

10.1128/msystems.01450-21.3 FIG S2 Temperature data logged over time (166 h) near the source of FC in 2008 (J. R. Havig, doctoral dissertation, Arizona State University, 2009). Download FIG S2, PDF file, 0.3 MB. Copyright © 2022 Hamilton and Havig. 2022 Hamilton and Havig. https://creativecommons.org/licenses/by/4.0/ This content is distributed under the terms of the Creative Commons Attribution 4.0 International license.

10.1128/msystems.01450-21.4 FIG S3 Temperature data logged over time (216 h) near the source of JJ in 2008 (J. R. Havig, doctoral dissertation, Arizona State University, 2009). Download FIG S3, PDF file, 1.6 MB. Copyright © 2022 Hamilton and Havig. 2022 Hamilton and Havig. https://creativecommons.org/licenses/by/4.0/ This content is distributed under the terms of the Creative Commons Attribution 4.0 International license.

**FIG 2:**
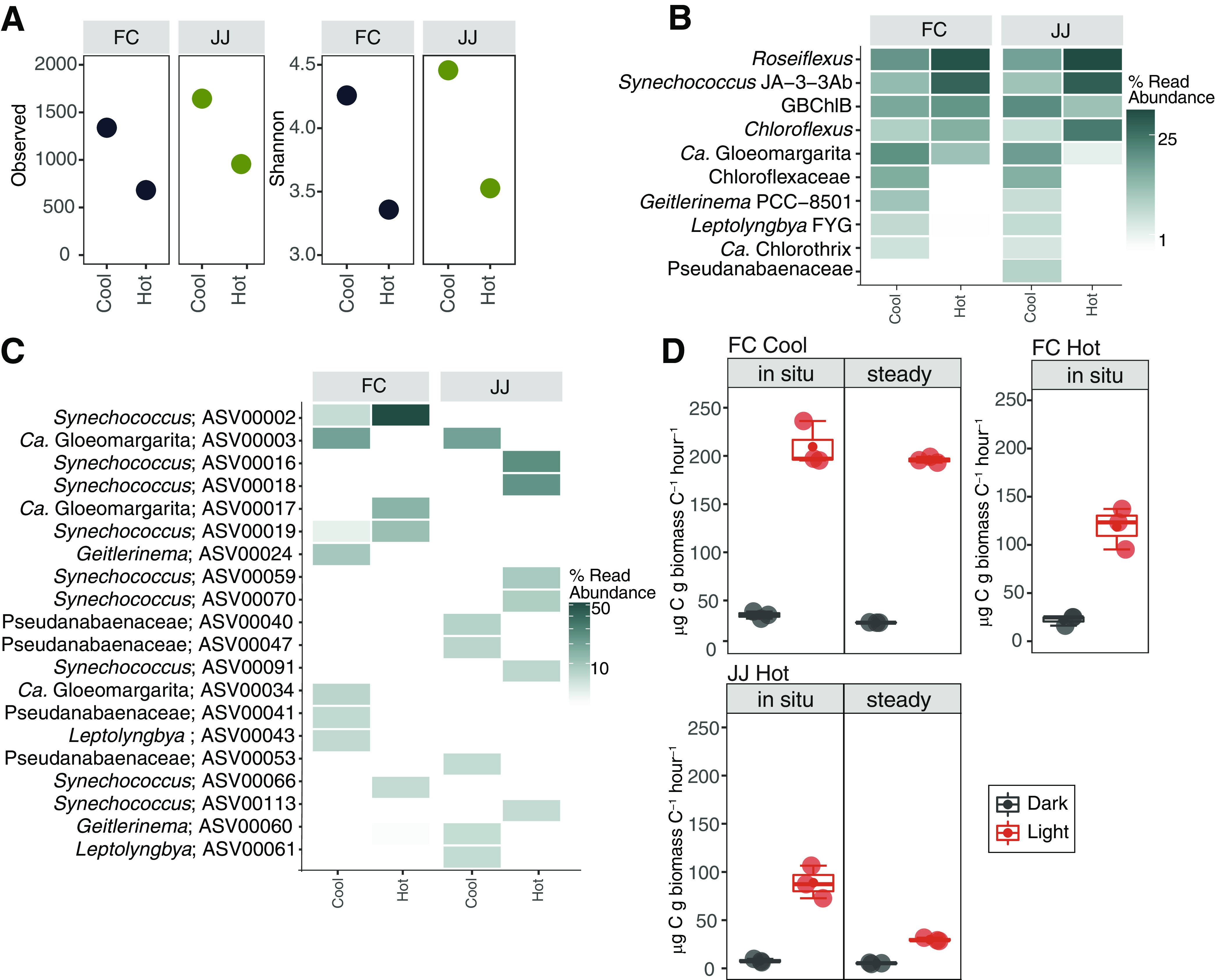
Diversity, phototroph community composition, and C assimilation rates. (A) Richness and Shannon diversity indices calculated for the 16S rRNA amplicons. (B) Heatmap of the relative abundance of OTUs assigned to putative bacterial phototrophs according to the work of Hamilton et al. (14). (C) Heatmap of the relative abundance of cyanobacterial ASVs. (D) Rates of C assimilation in microcosm assay performed in the dark (wrapped in foil) and light. Error bars from triplicate measurements. In all light-versus-dark comparisons, the rates are statistically different (*P *< 0.05). (Rates and *P* values are provided in Table S2.) Details of the methods are provided in Text S1.

Despite temperature fluctuations of up to 40°C, diversity and the composition of putative phototrophs in the geysing sites were similar to those in nongeysing sites (e.g., references 14 to 16): richness and diversity were lower in phototrophic mats near the upper temperature limit of photosynthesis (Fig. 2A), and at 97% sequence identity (defined as operational taxonomic units [OTUs]), sequences assigned to *Chloroflexi* (*Roseiflexus* and *Chloroflexus*), *Cyanobacteria* (*Synechococcus* and “*Candidatus* Gloeomargarita”), and *Chlorobi* (“*Candidatus* Thermochlorobacteriaceae bacterium GBChlB”) were abundant. Notably, sequences affiliated with other cyanobacteria, including “*Candidatus* Gloeomargarita,” *Geitlerinema* PCC-8501, *Leptolyngbya* FYG, and *Pseudanabaenaceae*, were recovered only from the “cool” sites, consistent with increasing diversity with decreasing temperature.

10.1128/msystems.01450-21.1 TEXT S1 Sample collection and aqueous geochemistry, CO_2_ assimilation (microcosms), nucleic acid extraction, 16S rRNA amplicon sequencing, sequence analysis, and data availability. Download Text S1, PDF file, 0.1 MB. Copyright © 2022 Hamilton and Havig. 2022 Hamilton and Havig. https://creativecommons.org/licenses/by/4.0/ This content is distributed under the terms of the Creative Commons Attribution 4.0 International license.

10.1128/msystems.01450-21.6 TABLE S2 Biomass C and N stable isotope analyses of biomass and rates of carbon assimilation and *P* values (for each comparison of C assimilation rates). All carbon isotope values are given as absolute values. All *P* values are <0.001. All samples have ^13^C-labeled bicarbonate added. Light, labeled bicarbonate added; dark, aluminum foil wrapped. St. dev., standard deviation of assays performed in triplicate. Download Table S2, PDF file, 0.06 MB. Copyright © 2022 Hamilton and Havig. 2022 Hamilton and Havig. https://creativecommons.org/licenses/by/4.0/ This content is distributed under the terms of the Creative Commons Attribution 4.0 International license.

Temperature selects for distinct cyanobacterial ecotypes in nongeysing outflows (e.g., A′ and A ecotypes occur at higher temperatures while B′ and B are observed at lower temperatures [17]). However, in our geysing outflows, all but one of the most abundant *Synechococcus* cyanobacterial ecotypes (identified as amplicon sequence variants [ASVs]) shared the highest sequence identity with the B′ ecotype. This indicates that median temperature (e.g., 56.0°C at FC hot and 61.5°C at JJ hot) drives ecotype differentiation in fluctuating systems despite regular exposure to higher temperatures that select for distinct ecotypes in nongeysing systems (e.g., A′ and A ecotypes [17]). With a few exceptions (e.g., ASV00002 and ASV00003), the ASVs from JJ and FC were distinct from each other while ASVs from “hot” and “cool” sites within the same hot spring outflow were also distinct (Fig. 2C). These data are consistent with a role for both geographic isolation and temperature in driving diversification and provide a framework to further examine allopatry versus physical and chemical characteristics in driving cyanobacterial evolution and diversification under continuously fluctuating temperatures.

We hypothesized that relatively stable temperatures at FC would result in higher rates of photoautotrophy (based on light-dependent C assimilation rates) compared to JJ and that the large fluctuation in temperatures at both would result in lower photoautotrophy rates compared to steady-temperature sites. We performed microcosm assays by placing mats and water from hot and cool sites at FC or JJ in sealed serum vials that were amended with NaH^13^CO_3_ following the methods in reference 14. To test our hypotheses, vials were incubated under the following conditions: (i) “*in situ*”—vials placed at the sample location, experiencing fluctuating temperatures (Fig. 1); (ii) “steady”—vials placed in nearby noneruptive hot springs meant to mimic lower temperatures observed at each site (FC cool and JJ hot). As expected, *in situ* rates were higher at FC than at JJ (Fig. 2D). For *in situ* versus steady, the C assimilation rate for the JJ hot mat held at a steady low temperature (steady in Fig. 2D; 28.1 μg C uptake/g C biomass/h) was lower than that for *in situ* microcosms while the C assimilation rates between *in situ* and steady treatments at FC cool were indistinguishable. Overall, light-dependent C assimilation rates at both eruptive sites were lower than rates observed for alkaline phototrophic communities collected from springs with similar temperature and pH in YNP (14, 15). For example, in previous studies of phototrophic mats, filaments, and biofilms from nongeysing alkaline hot springs with similar pHs in YNP (e.g., pH 7 to 9), observed light-dependent C assimilation rates ranged from 658.3 to 3813.8 μg C uptake/g C biomass/h (14, 15).

We propose that eruptive hot springs are an overlooked but key ecosystem for examining outstanding questions regarding the ecophysiology of hot spring cyanobacteria including whether adaptation to increasingly higher temperatures results in narrowing niche breadth (3, 18), the roles of temperature and allopatry in driving diversification, and how *Cyanobacteria* adapt to high, fluctuating temperatures. Our data indicate stable temperatures might drive higher fitness: light-dependent C assimilation rates were higher at FC which, while more chaotic in eruption periodicity, supported outflows with stable temperatures 68% of the time compared to more regular eruptivity but continuous temperature variation observed at JJ (changing discharge ∼54% of the time). In addition, we recovered sequences most closely related to B′, a lower-temperature cyanobacterial ecotype, across a broad niche breadth (at least in terms of temperature). Thus, while median rather than maximum temperature appears to drive cyanobacterial diversification in geysing outflows, the full range of adaptation to high temperature in hot spring *Synechococcus*, particularly in ecotypes from geysing systems, warrants further investigation. Indeed, there is rich history of previous studies on cyanobacterial ecotypes and thus an established comparative framework for examining the evolutionary history and ecophysiology of ecotypes in geysing systems through characterization of new isolates and genomic and metagenomics approaches.

### Data availability.

All sequence data including raw reads with quality scores for this study have been deposited in the NCBI Sequence Read Archive (SRA) database under the BioProject number PRJNA756970. Library designations are provided in Table S3.

10.1128/msystems.01450-21.7 TABLE S3 Accession numbers for the 16S rRNA amplicon libraries included in the present study. Download Table S3, PDF file, 0.04 MB. Copyright © 2022 Hamilton and Havig. 2022 Hamilton and Havig. https://creativecommons.org/licenses/by/4.0/ This content is distributed under the terms of the Creative Commons Attribution 4.0 International license.
