# Supplementary figures and images for: Meet Me in the Middle: Median Temperatures Impact Cyanobacteria and Photoautotrophy in Eruptive Yellowstone Hot Springs
Source: mSystems. 2022 Jan 4;7(1):e01450-21. doi: 10.1128/msystems.01450-21 (PMC8725584; doi:10.1128/msystems.01450-21)

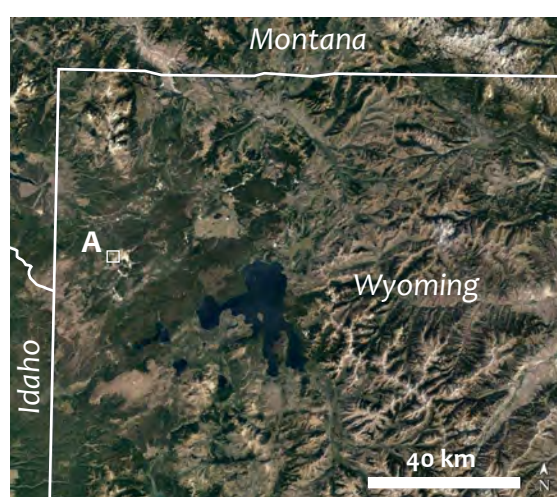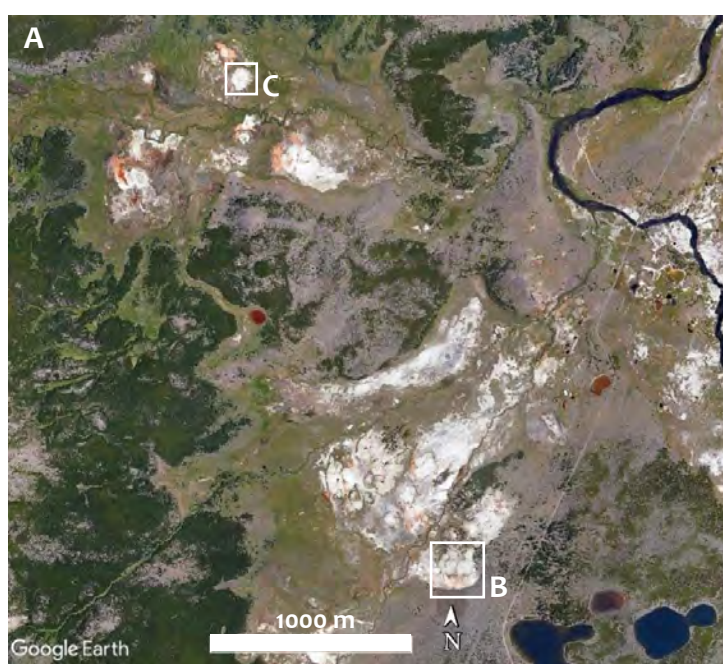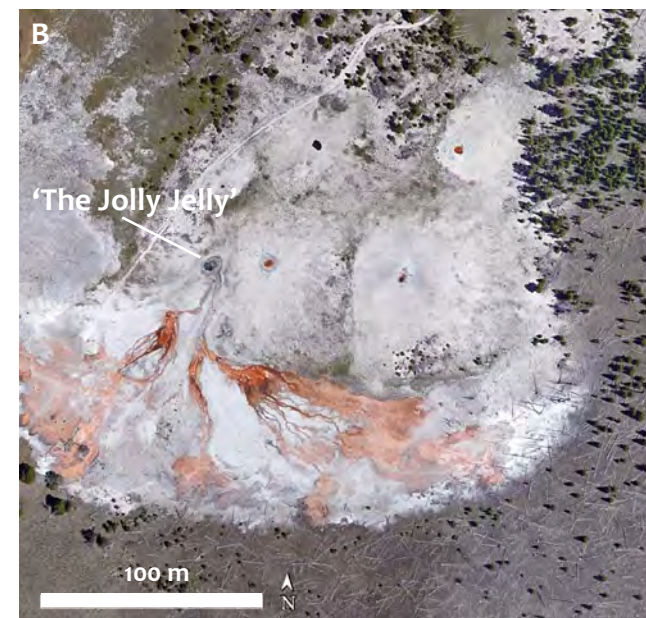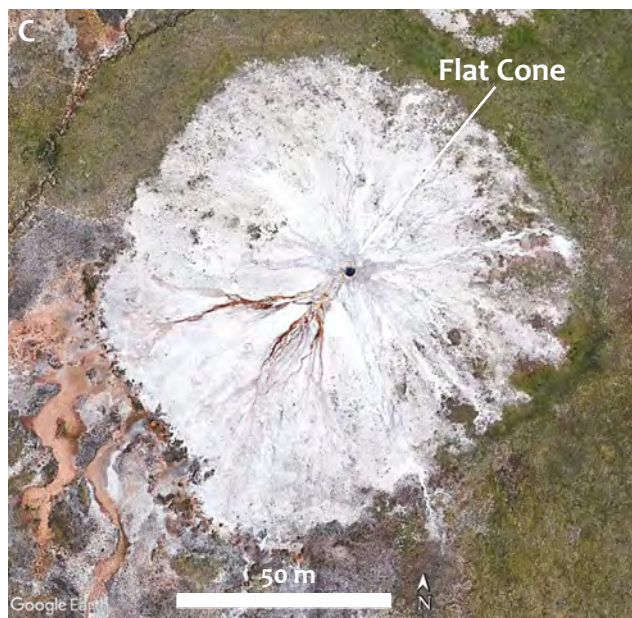

Supplement: FIG S1 [file msystems.01450-21-sf001.pdf]
