# Supplementary material for: Meet Me in the Middle: Median Temperatures Impact Cyanobacteria and Photoautotrophy in Eruptive Yellowstone Hot Springs
Source: mSystems. 2022 Jan 4;7(1):e01450-21. doi: 10.1128/msystems.01450-21 (PMC8725584; doi:10.1128/msystems.01450-21)
Supplement: TABLE S1 [file msystems.01450-21-st001.pdf]

				GPS											DIC		DOC	
				12 T	UTM	Elevation												
Site	pH	Temp (°C)	Cond. (µS/cm)	Easting	Northing	(m)	NO_3^- (µM)	NH_4(T) (µM)	Sulf. (µM)	Fe^{2+} (µM)	SiO_2 (mm)	Cl^- (mM)	SO_4^{2-} (mM)	PO_4^{3-} (µM)	mM	$\delta^{13}C_{VPDB}$ (‰)	µmol/ L	$\delta^{13}C_{VPDB}$ (‰)
FC cool	8.93	46.4	2104	510826	4935019	2202	bdl	10.0	0.56	bdl	5.08	6.43	0.16	0.15	3.91	-1.90	22.32	-24.70
FC hot	8.63	66.5	2786	510821	4935021	2202	14.3	7.1	0.59	bdl	5.83	6.29	0.16	0.21	4.06	-2.26	59.32	-24.46
JJ cool	9.09	48.3	2786	511960	4932577	2228	14.3	7.1	0.47	0.18	3.42	9.64	0.25	0.22	5.62	0.09	73.57	-24.59
JJ hot	8.91	70.0	3687	511905	4932605	2229	14.3	8.6	0.34	0.18	4.26	9.18	0.24	0.21	5.78	-0.61	34.79	-25.02
