# Supplementary material for: Meet Me in the Middle: Median Temperatures Impact Cyanobacteria and Photoautotrophy in Eruptive Yellowstone Hot Springs
Source: mSystems. 2022 Jan 4;7(1):e01450-21. doi: 10.1128/msystems.01450-21 (PMC8725584; doi:10.1128/msystems.01450-21)

Flat Cone, Sentinel Meadows,
Lower Geyser Basin, Yellowstone National Park,
WY, USA

Total time: 166 hours
Steady flow: 68.3 %
No flow: 31.7 %

Total eruptions: 77
Average eruptions per day: 11.1
Median eruptive period: 106 minutes

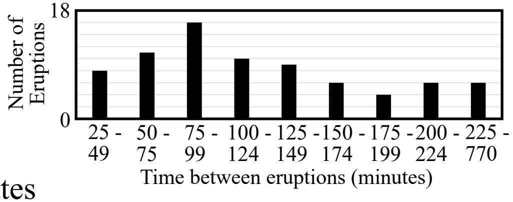

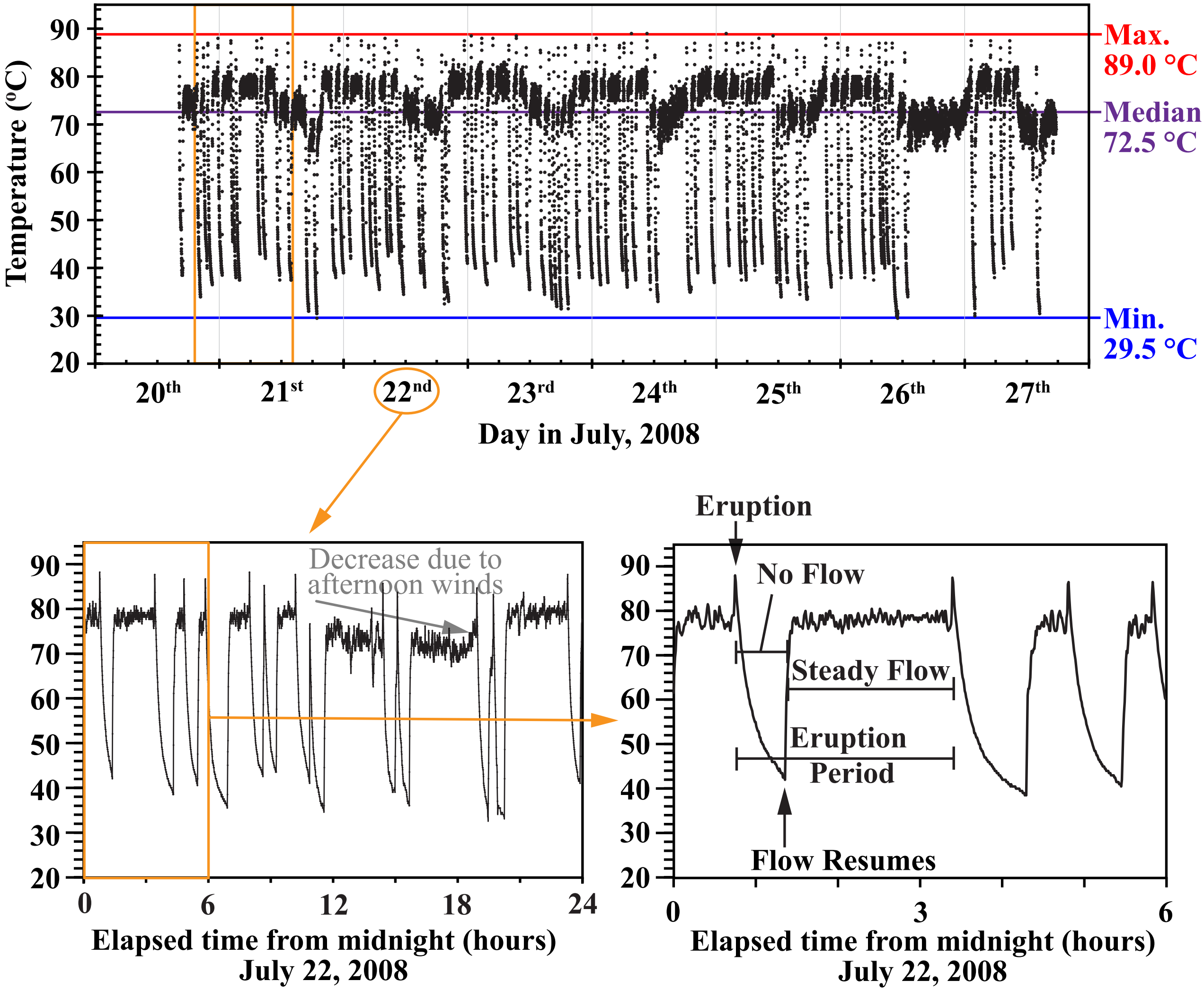

Supplement: FIG S2 [file msystems.01450-21-sf002.pdf]
