# Supplementary material for: Meet Me in the Middle: Median Temperatures Impact Cyanobacteria and Photoautotrophy in Eruptive Yellowstone Hot Springs
Source: mSystems. 2022 Jan 4;7(1):e01450-21. doi: 10.1128/msystems.01450-21 (PMC8725584; doi:10.1128/msystems.01450-21)

‘Jolly Jelly’, Imperial Geyser Basin,
Lower Geyser Basin, Yellowstone National Park,
WY, USA

Total time: 216 hours
Steady flow: 54.2 %
No flow: 45.8 %

Total eruptions: 147
Average eruptions per day: 16.3
Median eruptive period: 88 minutes

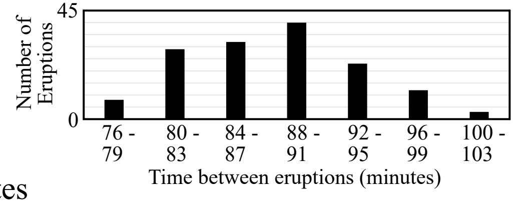

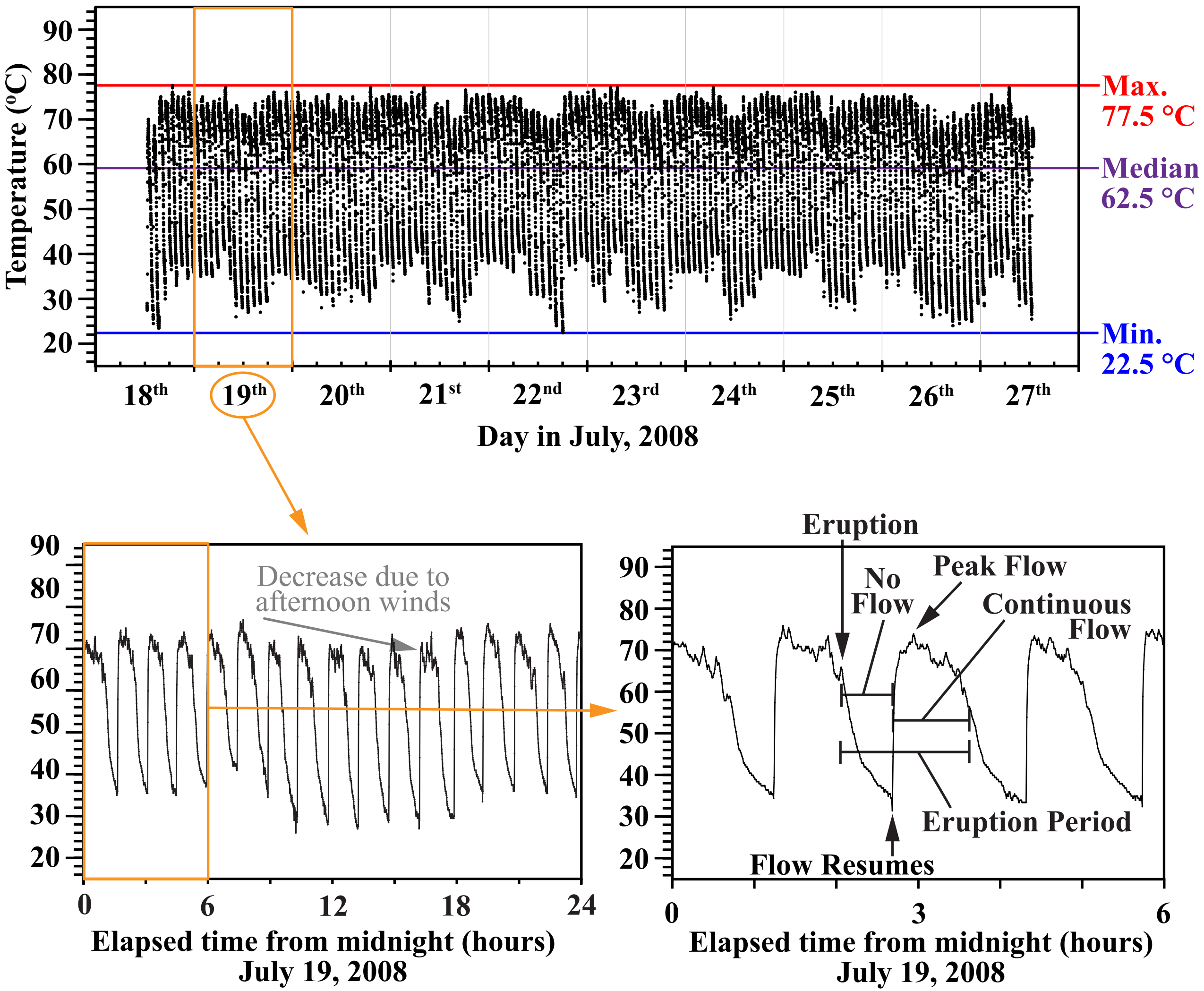

Supplement: FIG S3 [file msystems.01450-21-sf003.pdf]
