# Supplementary material for: Meet Me in the Middle: Median Temperatures Impact Cyanobacteria and Photoautotrophy in Eruptive Yellowstone Hot Springs
Source: mSystems. 2022 Jan 4;7(1):e01450-21. doi: 10.1128/msystems.01450-21 (PMC8725584; doi:10.1128/msystems.01450-21)
Supplement: TEXT S1 [file msystems.01450-21-s0001.pdf]

Supplemental Methods for

Meet me in the middle: median temperatures impact cyanobacteria and photoautotrophy in eruptive Yellowstone hot springs

Trinity L. Hamilton, Jeff R. Havig

Supplementary Methods

Sample collection and aqueous geochemistry

Samples were collected from geothermal features in Yellowstone National Park in August of 2019 (Table S1). Sample collection and aqueous geochemistry followed methods described previously (Hamilton et al., 2019). Sample locations and aqueous geochemistry are provided in Table S1. Temperature was logged over time using data loggers (Lascar Electronics EasyLog Thermocouple Data Loggers (EL-USB-TC-LCD)) with K-type thermocouples. Dataloggers were set to log temperatures every 30 seconds and to start logging upon depression of the logging button using the EasyLog software provided with the dataloggers. Dataloggers and thermocouples were deployed upon arrival at the sampling area with the thermocouple ends located at the sampling/incubation site, and were collected prior to departure from the sampling site at the end of the sampling day. Dataloggers were stopped and the data downloaded onto a laptop upon return to camp each day.

CO_2 assimilation (Microcosms)

The potential for inorganic carbon uptake *in situ* (C-assimilation) was assessed using microcosms through the addition of $NaH^{13}CO_3$ as described in Hamilton et al., 2019. Briefly, ~300-mg of biomass and spring water from the collection site were placed into pre-combusted (12 h, 450°C) serum vials. Vials were capped with gas-tight butyl rubber septa and amended with $NaH^{13}CO_3$ (100 μM final concentration) (Cambridge Isotope Laboratories, Inc., Andover, MA, USA) using gas-tight syringe injection. Vials were exposed to light to assess the potential for photoautotrophic uptake and chemoautotrophic (dark) $NaH^{13}CO_3$ uptake was assessed in vials wrapped in foil. Care was taken to maintain *in situ* temperature and the structure of mats or filaments. All treatments were performed in triplicate. Reported values of ^{13}C-labelled DIC uptake (carbon fixation rates) reflect the difference in uptake between the biomass in the assays that received $NaH^{13}CO_3$ and the natural abundance biomass samples.

We were interested in comparing C-assimilation rates between sites (FC vs. JJ) and assessing if C-assimilation rates would be lower under fluctuating temperatures compared to a steady temperature. To examine C-assimilation rates between sites (FC vs. JJ), vials were incubated at the site of sample collection in each outflow channel. This treatment is referred to as '*in situ*' in Fig. 1 and Table S2. To examine if C-assimilation rates would be lower under fluctuating temperatures compared to a steady temperature, vials were incubated in near-by hot springs with steady outflow channels (non-eruptive sites). This treatment is referred to as 'steady' in Fig. 1 and Table S2. For the steady temperatures, we chose a temperature that was similar to the lower temperature in each site (38.8 °C for JJ and 40.5 °C for FC).

C stable isotope signals were determined in microcosms and natural abundance samples of biomass using a Costech Instruments Elemental Analyzer (EA) periphery connected to a Thermo Scientific Delta V Advantage Isotope Ratio Mass Spectrometer (IR-MS) at the UC Davis Stable Isotope Facility. For analyses, microcosm samples were thawed and biomass was rinsed with 1 M HCl to remove any extra ^{13}C-labelled DIC, triple rinsed with 18.2 MΩ/cm deionized water, then dried (60°C for three days). Natural abundance samples were not treated with acid. Samples were ground/homogenized with a cleaned mortar and pestle (ground with ethanol silica slurry, triple rinsed with 18.2 MΩ/cm deionized water, dried before and between sample grinding), weighed, placed into tin boats, and sealed for analyses. To minimize the potential for minimize cross contamination, natural abundance samples and microcosm samples were processed and analyzed independently of label-exposed replicates. Standard checks and blanks were included in each run to check for memory effects or cross contamination of samples, with none detected. C and N stable isotope analyses are provided in Table S2.

Inorganic carbon assimilation rates were calculated from the total µg C taken up (difference between ^{13}C content of natural abundance sample and label-exposed replicates) divided by the grams of organic C per gram of sediment and the number of hours the incubation was carried out over (typically ~ 2 hours). Mean ^{13}C uptake rates were compared using a one-way ANOVA followed by *post hoc* pairwise comparisons between treatments was conducted using a Tukey honest significant difference (HSD) within the R software package (R version 4.0.2). Mean rates with p-values < 0.05 were considered significantly different. Rates and p-values are provided in Table S2.

Nucleic acid extraction and 16S rRNA amplicon sequencing

Total DNA was extracted from triplicate ~250 mg samples using a DNeasy PowerSoil Kit (Qiagen, Carlsbad, CA, USA) according to the manufacturer's instructions. Triplicate samples were pooled and submitted to the University of Minnesota Genomics Center (UMGC) for 16S rRNA amplicon sequencing targeting the V4 hypervariable region of bacterial and archaeal 16S SSU rRNA gene using the primers 515F and 806R following the methods in Gohl et al 2016. Amplicons were sequenced using MiSeq Illumina 2 × 300 bp chemistry. Each sample was sequenced once. DNA was extracted from a 0.2 µm polyethersulfone syringe filters (VWR International, Radnor, PA, USA) used for the field blank water sample (described in the SOM) as a negative DNA extraction control. No DNA was in the filter controls and sequencing failed to generate amplicons.

Sequence analysis

Post sequence processing was performed using mothur (ver. 1.45.3) following the MiSeq SOP (Schloss et al., 2009; Kozich et al., 2013). Read pairs were assembled into contigs and trimmed to include only the overlapping regions. Unique sequences were aligned and classified using a Bayesian classifier and against

a SILVA-based reference alignment (v138) and the SILVA reference taxonomy (v138), respectively. Chimeras were identified in the aligned sequences using UCHIME (Edgar et al., 2011) and removed from further analyses. Sequences were binned into operational taxonomic units (OTUs) based on a sequence similarity of 97.0%. OTUs assigned to mitochondria or chloroplast were removed and not included in the rest of the analyses. Following quality control, merging of contigs, and removal of chimeras and singletons, we recovered 143,413 16S rRNA gene sequences with an average length of ~250 bp. The libraries ranged in size from 32,297 to 40,669 total sequences. At a sequence identity of 97%, we recovered 3,527 total OTUs. Amplicon sequence variants (ASVs) were identified using mothur and those affiliated with *Synechococcus* ("*Leptococcus*" in the Silva database) were selected for further analyses. ASV sequences assigned to *Synechococcus* were subjected to BlastN searches to identify the closest hit. In addition, taxonomy of the ASV sequences assigned to *Synechococcus* were examined using the SILVA Alignment, Classification and Tree (ACT) Service (www.arb-silva.de/act). The ASV sequences and closely related sequences were aligned using SINA aligner (Pruesse et al., 2012) and a phylogenetic tree was reconstructed using RAxML (Stamatakis 2014). Trees were visualized using iTOL (Letunic and Bork 2016). Analyses and visualization of the amplicon was carried out using R (R version 4.0.2) including the Phyloseq (ver. 1.16.2; McMurdie and Holmes, 2013) and ampvis2 (Anderson et al., 2018) packages. For analysis of alpha diversity, each sample was rarefied to an even depth.

Data availability

All sequence data including raw reads with, quality scores for this study have been deposited in the NCBI Sequence Read Archive (SRA) database under with the BioProject number PRJNA756970. Library designations are provided in Table S3.

References

Andersen KS, Kirkegaard RH, Karst SM, Albertsen, M. (2018). ampvis2: an R package to analyse and visualise 16S rRNA amplicon data. *bioRxiv* 2018. 10.1101/299537. (doi: https://doi.org/10.1101/299537)

Edgar RC, Haas BJ, Clemente JC, Quince C, Knight R. (2011). UCHIME improves sensitivity and speed of chimera detection. *Bioinformatics* **27**:2194–2200. (doi:10.1093/bioinformatics/btr381)

Gohl DM, Vangay P, Garbe J, MacLean A, Hauge A, Becker A, Gould TJ, Clayton JB, Johnson TJ, Hunter R, Knights D, Beckman KB. (2016). Systematic improvement of amplicon marker gene methods for increased accuracy in microbiome studies. *Nat Biotechnol* **34**:942. (doi:10.1038/nbt.3601)

Hamilton TL, Bennett AC, Murugapiran S, Havig JR (2019). Anoxygenic phototrophs span geochemical gradients and diverse morphologies in terrestrial geothermal springs. *mSystems* 4:e00498-19. (doi: 10.1128/mSystems.00498-19)

Havig JR. (2009). Geochemistry of hydrothermal biofilms: Composition of biofilms in siliceous sinter-depositing hot springs, doctoral dissertation, School of Earth and Space Exploration, Ariz. State Univ., Tempe.

Kozich JJ, Westcott SL, Baxter NT, Highlander SK, Schloss PD. (2013). Development of a dual-index sequencing strategy and curation pipeline for analyzing amplicon sequence data on the MiSeq Illumina sequencing platform. *Appl Environ Microbiol* **79**:5112–5120. (doi:10.1128/AEM.01043-13)

Letunic I, Bork P. (2016). Interactive tree of life (iTOL) v3: an online tool for the display and annotation of phylogenetic and other trees. *Nucleic Acids Res* **44**:W242–5. (doi:10.1093/nar/gkw290)

McMurdie PJ, Holmes S. (2013). phyloseq: an R package for reproducible interactive analysis and graphics of microbiome census data. *PLoS One* **8**: e61217. (doi:10.1371/journal.pone.0061217)

Pruesse E, Peplies J, Glöckner FO. (2012). SINA: accurate high-throughput multiple sequence alignment of ribosomal RNA genes. *Bioinformatics* **28**:1823–1829. (doi:10.1093/bioinformatics/bts252)

Schloss PD, Westcott SL, Ryabin T, Hall JR, Hartmann M, Hollister EB, et al. (2009). Introducing mothur: open-source, platform-independent, community-supported software for describing and comparing microbial communities. *Appl Environ Microbiol* **75**:7537–7541. (doi:10.1128/AEM.01541-09)

Stamatakis A. (2014). RAxML Version 8: A tool for Phylogenetic Analysis and Post-Analysis of Large Phylogenies. *Bioinformatics* **30**:1312-3. (doi:10.1093/bioinformatics/btu033)
