# Supplementary material for: Meet Me in the Middle: Median Temperatures Impact Cyanobacteria and Photoautotrophy in Eruptive Yellowstone Hot Springs
Source: mSystems. 2022 Jan 4;7(1):e01450-21. doi: 10.1128/msystems.01450-21 (PMC8725584; doi:10.1128/msystems.01450-21)
Supplement: TABLE S2 [file msystems.01450-21-st002.pdf]

Site			Biomass			
		Total C (%)	$\delta^{13}C$ (‰)	Total N (%)	$\delta^{15}N$‰	
FC cool		12.87	-17.64	0.35	-0.70	
FC hot		3.16	-19.22	1.14	3.74	
JJ cool		9.51	-17.38	0.81	-1.79	
JJ hot		1.75	-19.31	0.18	*-0.90*	

			Microcosms		
Site	Treatment		μg C/g biomass/hr	St. dev.	*p*-value
FC cool	in situ	Light	189.57	23.10	0.000
		Dark	14.98	3.69	
	steady	Light	204.67	46.32	0.000
		Dark	7.12	0.22	
FC hot		Light	118.66	21.44	0.001
		Dark	21.98	4.96	
JJ hot	in situ	Light	88.80	16.95	0.001
		Dark	7.82	1.79	
	steady	Light	28.09	1.77	0.000
		Dark	3.51	0.63	
