# Supplementary material for: Meet Me in the Middle: Median Temperatures Impact Cyanobacteria and Photoautotrophy in Eruptive Yellowstone Hot Springs
Source: mSystems. 2022 Jan 4;7(1):e01450-21. doi: 10.1128/msystems.01450-21 (PMC8725584; doi:10.1128/msystems.01450-21)
Supplement: TABLE S3 [file msystems.01450-21-st003.pdf]

Site	Library Name	Accession number
FC cool	803G	SAMN20931015
FC hot	803H	SAMN20931016
JJ cool	802A	SAMN20931013
JJ hot	802Y	SAMN20931014
